# Notes on the Treatment of Bronchitis

**Published:** 1896-05-30

**Authors:** Solomon C. Smith

**Affiliations:** Physician to the Westminster General Infirmary


### Medical Progress and Hospital Clinics.

[*The Editor will be glad to receive offers of co-operation and contributions from members of the profession. All letters should be addressed to* The Editor, at the Office, 428, Strand, London, W.C.]

### NOTES ON THE TREATMENT OF BRONCHITIS.

By Solomon C. Smith, M.D., M.R.C.P., Physician to the Westminster General Infirmary.

The changes which take place both in the air and the blood within the lungs during the act of respiration are so marked, and the lungs are so obviously necessary to life, that one is apt by contrast to look upon the bronchi as organs of but small functional activity. A tube, in fact, would seem to fulfil its purpose by merely remaining open, and thus the bronchi are too often regarded as merely passive channels.

A study of the phenomena which so often precede and accompany the onset of death should, however, be sufficient to dissipate any such idea. Anyone who will carefully observe the development of the "death rattle," and will compare what happens then, when the air tubes are really passive, with what goes on in ordinary healthy breathing, will see at once that the bronchi are fairly active partners in the act of respiration. In the one case the accumulating mucus is churned backwards and forwards, but is not expelled, while in the other it is steadily driven towards the laryngeal outlet by the activity of the ciliated epithelium of the tubes and by their muscular contractility.

Any foreign substance, then (and an accumulation of mucus is a foreign substance), tends in the living tube to be driven outwards instead of being pushed backwards and forwards, and one of the most important effects of bronchitis is the diminution of this expelling or self-cleansing power, and the reduction of the tube towards that passive condition which is seen in its extreme degree when death is imminent.

In the assemblage of symptoms, then, which characterise an attack of bronchitis those which arise from the mere presence of inflammation of the mucus membrane are to a large extent lost amid those which depend on the interference of that inflammation with the function of the tube. Partly as the result of the diminished activity of the ciliæ, partly from the lessened activity of the muscular layer, and partly, as the case proceeds into the chronic stage, by the thinning and loss of elasticity which takes place in the whole structure, and the occurrence of local dilatations or bronchiectases, the self-cleansing power of the tube diminishes, while as the direct result of the inflammation the secretion increases in quantity and becomes more adhesive and more difficult of removal.

One of the earliest effects of inflammation of the bronchial tubes is shown in its influence upon the secretion by which, in health, the mucous membrane is kept at a proper degree of moisture. Instead of a clear and simple fluid, this becomes a thick and adhesive mucous, crowded with cells, which, in turn, are often crowded with bacteria, and it accumulates in masses which are quite beyond the power of the normal expulsive apparatus, and can only be removed by coughing. Every bronchus is surrounded by—one may almost say lies embedded in—a mass of lymphoid tissue, as well as by mucous glands, and all these are thrown into a state of great activity whenever the mucous surface is irritated or inflamed. We see, then, that the effect of inflammation of the bronchial tubes, besides lessening their self-cleansing power, is to increase greatly the amount of *débris* which has to be removed, to cause great activity and congestion, and therefore swelling, of the substance of the tubes and the parts underlying them, and thus, as a first effect, to cause narrowing of their lumen, and, in consequence, difficulty of breathing, and later, by its continuance or repetition, to produce such permanent changes in their structure as to interfere with their function, so that, after a few attacks of bronchitis, it is too often the case that the patient is left either a permanent sufferer, or liable to recurrences of the disease on exposure to comparatively trifling causes.

There is, however, another thing to be considered before we can fully understand the phenomena met with in bronchitis, viz., that the muscular layer by which the finer bronchi are surrounded is liable to pass into a state of spasmodic contraction when the mucous surface is irritated.

In its independent form, that is when occurring as a reflex from some other organ, this is ordinary asthma, but it is perfectly well recognised that nothing is more likely to give rise to such spasm of the bronchioles than inflammation of the bronchial mucous membrane. No small portion then of the dyspnœa experienced in many cases of bronchitis arises from spasm of the tubes, which may not only turn a comparatively simple case into one of dangerous severity, but may, as a set off, sometimes enable a practitioner who recognises the position of affairs to give striking relief in what has appeared a condition of great danger—not, perhaps, by curing the bronchitis, but by lessening the superadded spasm and letting the air again into the lungs.

In regard to the effects produced by bronchitis on other organs, it may be said that except for the muscular fatigue and loss of sleep produced by the cough and dyspnœa, and for the septic influence of the abnormal bronchial secretion, they are limited to the heart and lungs, the obstruction in the bronchi leading to emphysema and collapse of the lungs, with lobular pneumonia, while the impediment to the pulmonary circulation produced by these changes leads to dilatation and hypertrophy of the right side of the heart. It thus not infrequently happens that patients affected with bronchitis also suffer from the want of breath and the imperfect aeration of the blood which accompany emphysema, and from the dropsy and general failure which comes on when the heart is no longer able to furnish the tissues with their normal supply of blood.

